# Claude 3 Opus and ChatGPT With GPT-4 in Dermoscopic Image Analysis for Melanoma Diagnosis: Comparative Performance Analysis

**DOI:** 10.2196/59273

**Published:** 2024-08-06

**Authors:** Xu Liu, Chaoli Duan, Min-kyu Kim, Lu Zhang, Eunjin Jee, Beenu Maharjan, Yuwei Huang, Dan Du, Xian Jiang

**Affiliations:** 1 Department of Dermatology West China Hospital Sichuan University Chengdu China

**Keywords:** artificial intelligence, AI, large language model, LLM, Claude, ChatGPT, dermatologist

## Abstract

**Background:**

Recent advancements in artificial intelligence (AI) and large language models (LLMs) have shown potential in medical fields, including dermatology. With the introduction of image analysis capabilities in LLMs, their application in dermatological diagnostics has garnered significant interest. These capabilities are enabled by the integration of computer vision techniques into the underlying architecture of LLMs.

**Objective:**

This study aimed to compare the diagnostic performance of Claude 3 Opus and ChatGPT with GPT-4 in analyzing dermoscopic images for melanoma detection, providing insights into their strengths and limitations.

**Methods:**

We randomly selected 100 histopathology-confirmed dermoscopic images (50 malignant, 50 benign) from the International Skin Imaging Collaboration (ISIC) archive using a computer-generated randomization process. The ISIC archive was chosen due to its comprehensive and well-annotated collection of dermoscopic images, ensuring a diverse and representative sample. Images were included if they were dermoscopic images of melanocytic lesions with histopathologically confirmed diagnoses. Each model was given the same prompt, instructing it to provide the top 3 differential diagnoses for each image, ranked by likelihood. Primary diagnosis accuracy, accuracy of the top 3 differential diagnoses, and malignancy discrimination ability were assessed. The McNemar test was chosen to compare the diagnostic performance of the 2 models, as it is suitable for analyzing paired nominal data.

**Results:**

In the primary diagnosis, Claude 3 Opus achieved 54.9% sensitivity (95% CI 44.08%-65.37%), 57.14% specificity (95% CI 46.31%-67.46%), and 56% accuracy (95% CI 46.22%-65.42%), while ChatGPT demonstrated 56.86% sensitivity (95% CI 45.99%-67.21%), 38.78% specificity (95% CI 28.77%-49.59%), and 48% accuracy (95% CI 38.37%-57.75%). The McNemar test showed no significant difference between the 2 models (*P*=.17). For the top 3 differential diagnoses, Claude 3 Opus and ChatGPT included the correct diagnosis in 76% (95% CI 66.33%-83.77%) and 78% (95% CI 68.46%-85.45%) of cases, respectively. The McNemar test showed no significant difference (*P*=.56). In malignancy discrimination, Claude 3 Opus outperformed ChatGPT with 47.06% sensitivity, 81.63% specificity, and 64% accuracy, compared to 45.1%, 42.86%, and 44%, respectively. The McNemar test showed a significant difference (*P*<.001). Claude 3 Opus had an odds ratio of 3.951 (95% CI 1.685-9.263) in discriminating malignancy, while ChatGPT-4 had an odds ratio of 0.616 (95% CI 0.297-1.278).

**Conclusions:**

Our study highlights the potential of LLMs in assisting dermatologists but also reveals their limitations. Both models made errors in diagnosing melanoma and benign lesions. These findings underscore the need for developing robust, transparent, and clinically validated AI models through collaborative efforts between AI researchers, dermatologists, and other health care professionals. While AI can provide valuable insights, it cannot yet replace the expertise of trained clinicians.

## Introduction

Artificial intelligence (AI) has shown great promise in various medical fields, including dermatology [1]. The emergence of large language models (LLMs), such as ChatGPT, has demonstrated their ability to generate human-like responses and assist in clinical decision-making [2]. With the introduction of image analysis capabilities in LLMs [3], their application in dermatological diagnostics has attracted significant interest. Claude 3 Opus, an advanced conversational AI model, has shown promising performance in various natural language processing tasks [4]. This study aims to fill that gap by comparing Claude 3 Opus and ChatGPT with GPT-4. Despite the potential of AI in health care, encompassing diagnosis, treatment, and public health initiatives, these technologies are largely underused in clinical practice [5]. Moreover, the use of AI in health care raises important legal and ethical considerations, particularly for “high-risk” applications such as medical diagnosis [6]. In this context, comparing the diagnostic performance of Claude 3 Opus and ChatGPT can provide valuable insights into their strengths and limitations, guiding the selection and optimization of AI-assisted diagnostic tools in dermatology while taking into account the regulatory landscape.

## Methods

**Figure 1:**
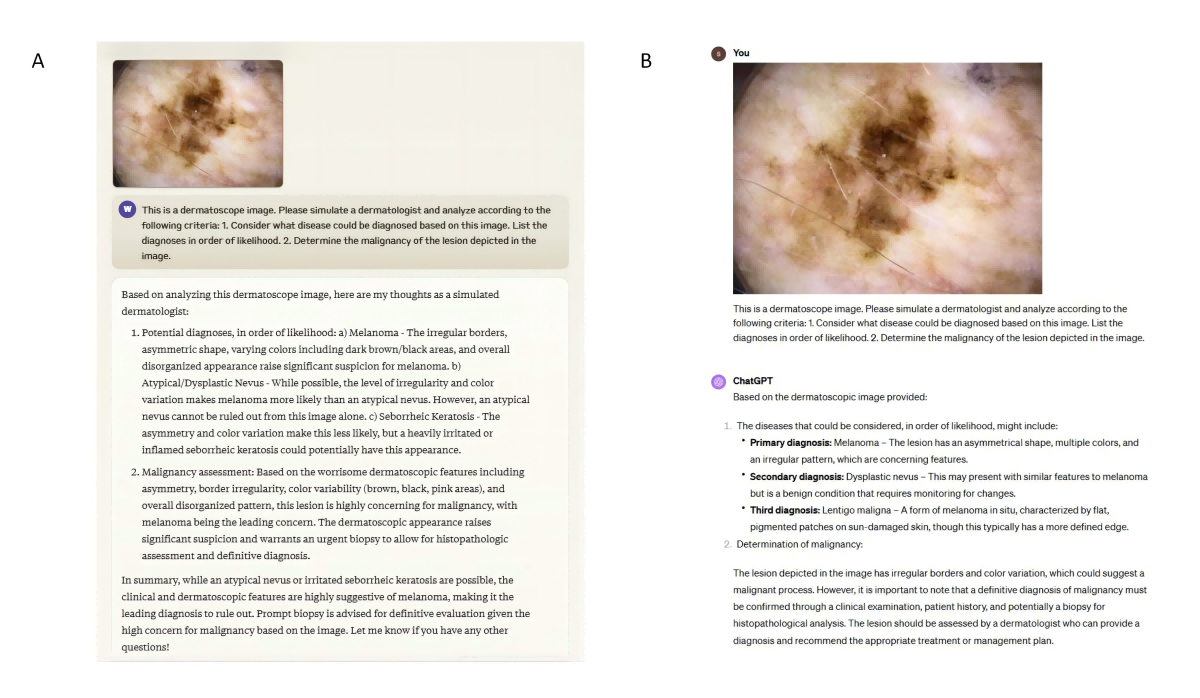
Performance comparison of Claude 3 Opus and ChatGPT with GPT4-Vision in skin dermoscopy image analysis and melanoma diagnosis: application scenarios. (A.) Application scenario of Claude 3 Opus in the analysis process of dermoscopic images. (B) Application scenario of GPT4-Vision in the analysis process of dermoscopic images.

We randomly selected 100 dermoscopic images (50 malignant melanomas, 50 benign nevi) from the International Skin Imaging Collaboration (ISIC) archive [7] using a computer-generated randomization process to avoid selection bias. The ISIC archive was chosen due to its comprehensive and well-annotated collection of dermoscopic images, which ensures a diverse and representative sample. Images were included if they were dermoscopic images of melanocytic lesions with histopathologically confirmed diagnoses. Each image was presented to Claude 3 Opus and ChatGPT. The models were given the same prompt, instructing them to provide the top 3 differential diagnoses for each image, ranked by likelihood. The exact prompt was “Please provide the top 3 differential diagnoses for this dermoscopic image, ranked by likelihood. Focus on distinguishing between melanoma and benign nevi.” The models’ responses were recorded for analysis (Figure 1A, B). We assessed primary diagnosis accuracy, accuracy of the top 3 differential diagnoses, and malignancy discrimination ability. The McNemar test was used to compare the models' performance.

## Results

For the primary diagnosis, Claude 3 Opus achieved 54.9% sensitivity (95% CI 44.08%-65.37%), 57.14% specificity (95% CI 46.31%-67.46%), and 56% accuracy (95% CI 46.22%-65.42%), while ChatGPT demonstrated 56.86% sensitivity (95% CI 45.99%-67.21%), 38.78% specificity (95% CI 28.77%-49.59%), and 48% accuracy (95% CI 38.37%-57.75%). The McNemar test showed no significant difference between the 2 models (*P*=.17; Multimedia Appendices 1-2). For the top 3 differential diagnoses, Claude 3 Opus and ChatGPT included the correct diagnosis in 76% (95% CI 66.33%-83.77%) and 78% (95% CI 68.46%-85.45%) of cases, respectively. The McNemar test showed no significant difference (*P*=.56). In malignancy discrimination, Claude 3 Opus outperformed ChatGPT with 47.06% sensitivity, 81.63% specificity, and 64% accuracy, compared to 45.1%, 42.86%, and 44%, respectively. The McNemar test showed a significant difference (*P*<.001). Claude 3 Opus had an odds ratio of 3.951 (95% CI 1.685-9.263) in discriminating malignancy, while ChatGPT had an odds ratio of 0.616 (95% CI 0.297-1.278) (Multimedia Appendix 3).

## Discussion

Our study demonstrates the potential of LLMs in assisting dermatological diagnosis, while also revealing their current limitations. Claude 3 Opus showed superior performance in discriminating between malignant and benign lesions compared to ChatGPT. However, both models made errors in diagnosing melanoma and nevi. For example, Claude 3 Opus misdiagnosed several melanomas as benign lesions, while ChatGPT had a higher false positive rate, misclassifying many nevi as melanomas. These findings highlight the need for further development and rigorous clinical validation of AI diagnostic tools before their widespread implementation in dermatology practice (Multimedia Appendix 4). Future research should focus on improving the robustness and interpretability of these models through close collaboration between AI researchers, dermatologists, and other health care stakeholders. Moreover, the potential impact of AI in health care extends beyond technical performance, encompassing legal and ethical dimensions. The European Commission has already proposed legislation for “high-risk” AI applications, providing a framework for the safe and responsible use of medical AI [6]. As LLMs and other AI tools continue to advance, it is crucial to proactively address these regulatory aspects to ensure their beneficial integration into clinical practice. In conclusion, while LLMs such as Claude 3 Opus and ChatGPT show promise in assisting dermatological diagnosis, they are not yet capable of replacing human expertise. Continued research, collaborative development, and proactive regulation are essential for realizing the full potential of AI in dermatology while prioritizing patient safety and ethical standards.

## Appendix

Multimedia Appendix 1 Claude 3 Opus and GPT4-Vision for the analysis of dermoscopic images and diagnosis of melanoma based on data comparison.

Multimedia Appendix 2 McNemar test results for the performance differences between Claude 3 Opus and GPT4-Vision.

Multimedia Appendix 3 Odds ratio and 95% CIs for Claude 3 Opus and GPT4-Vision for their ability to distinguish between benign and malignant conditions.

Multimedia Appendix 4 (A) Examples of Claude 3 Opus misdiagnosing melanoma as benign lesions. (B) Examples of Claude 3 Opus misdiagnosing benign lesions as melanoma. (C) Examples of GPT4-Vision misdiagnosing melanoma as benign lesions. (D) Examples of GPT4-Vision misdiagnosing benign lesions as melanoma.

## Abbreviations

AI: artificial intelligence
ISIC: International Skin Imaging Collaboration
LLM: large language model
