# Supplementary material for: Claude 3 Opus and ChatGPT With GPT-4 in Dermoscopic Image Analysis for Melanoma Diagnosis: Comparative Performance Analysis
Source: JMIR Med Inform. 2024 Aug 6;12:e59273. doi: 10.2196/59273 (PMC11336503; doi:10.2196/59273)
Supplement: Multimedia Appendix 1 [file medinform_v12i1e59273_app1.docx]

**Table 1.** Claude 3 Opus and GPT4-Vision in the analysis of dermoscopic images and the diagnosis of melanoma based on data comparison.

| **Performance Metric** | **Claude 3 Opus** | **GPT4-Vision** | **P** |
| --- | --- | --- | --- |
| Primary Diagnosis Accuracy |  |  |  |
| - Sensitivity（95% CI） | 54.90%  （40.34%-68.87%） | 56.86%  （42.26%-70.65%） | 0.170 |
| - Specificity（95% CI） | 57.14%  （42.21%-71.18%） | 38.78%  （25.13%-53.79%） |  |
| - Accuracy （95% CI） | 56.00%  （45.25%-66.30%） | 48.00%  （37.44%-58.73%） |  |
| Top 3 Differential Diagnoses Accuracy（95% CI） | 76.00%  （65.54%-84.65%） | 78.00%  （67.67%-86.35%） | 0.564 |
| Malignancy Discrimination Ability |  |  |  |
| - Sensitivity（95% CI） | 47.06%  （32.93%-61.54%） | 45.10%  （31.13%-59.61%） | 0.001 |
| - Specificity（95% CI） | 81.63%  （67.98%-91.24%） | 42.86%  （28.82%-57.79%） |  |
| - Accuracy （95% CI） | 64.00%  （53.19%-73.89%） | 44.00%  （33.70%-54.75%） |  |
