# Supplementary material for: Claude 3 Opus and ChatGPT With GPT-4 in Dermoscopic Image Analysis for Melanoma Diagnosis: Comparative Performance Analysis
Source: JMIR Med Inform. 2024 Aug 6;12:e59273. doi: 10.2196/59273 (PMC11336503; doi:10.2196/59273)
Supplement: Multimedia Appendix 2 [file medinform_v12i1e59273_app2.docx]

**Supplementary Table 1.** McNemar's test results for the performance differences between Claude 3 Opus and GPT4-Vision.

| **Performance Metric** | **Claude 3 Opus** | **GPT4-Vision** | **Discordant Pairs** | **McNemar's Chi-Squared Statistic** | ***P*** | **Significant Difference?** |
| --- | --- | --- | --- | --- | --- | --- |
| Primary Diagnosis Accuracy |  |  |  |  |  |  |
| - Correct | 56 | 48 | Claude 3 Opus correct, GPT4-Vision incorrect: 21 | 1.882 | 0.170 | No |
| - Incorrect | 44 | 52 | Claude 3 Opus incorrect, GPT4-Vision correct: 13 |  |  |  |
| Top 3 Differential Diagnoses Accuracy |  |  |  |  |  |  |
| - Correct | 76 | 78 | Claude 3 Opus correct, GPT4-Vision incorrect: 5 | 0.333 | 0.564 | No |
| - Incorrect | 24 | 22 | Claude 3 Opus incorrect, GPT4-Vision correct: 7 |  |  |  |
| Malignancy Discrimination Ability |  |  |  |  |  |  |
| - Correct | 64 | 44 | Claude 3 Opus correct, GPT4-Vision incorrect: 29 | 10.526 | 0.001 | Yes |
| - Incorrect | 36 | 56 | Claude 3 Opus incorrect, GPT4-Vision correct: 9 |  |  |  |
