# Supplementary material for: Claude 3 Opus and ChatGPT With GPT-4 in Dermoscopic Image Analysis for Melanoma Diagnosis: Comparative Performance Analysis
Source: JMIR Med Inform. 2024 Aug 6;12:e59273. doi: 10.2196/59273 (PMC11336503; doi:10.2196/59273)
Supplement: Multimedia Appendix 3 [file medinform_v12i1e59273_app3.docx]

**Supplementary Table 2.** The OR values and their 95% confidence intervals for Claude 3 Opus and GPT4-Vision in terms of their ability to distinguish between benign and malignant conditions.

| **Model** | **TP** | **FP** | **FN** | **TN** | ***OR*** | **95% CI** |
| --- | --- | --- | --- | --- | --- | --- |
| **Claude 3 Opus** | 24 | 9 | 27 | 40 | 3.951 | [1.685, 9.263] |
| **GPT4-Vision** | 23 | 28 | 28 | 21 | 0.616 | [0.297, 1.278] |

* OR: Odds Ratio, CI: Confidence Interval, TP: True Positive (model predicted malignant, actually malignant), FP: False Positive (model predicted malignant, actually benign), FN: False Negative (model predicted benign, actually malignant), TN: True Negative (model predicted benign, actually benign).
