# Supplementary material for: Claude 3 Opus and ChatGPT With GPT-4 in Dermoscopic Image Analysis for Melanoma Diagnosis: Comparative Performance Analysis
Source: JMIR Med Inform. 2024 Aug 6;12:e59273. doi: 10.2196/59273 (PMC11336503; doi:10.2196/59273)
Supplement: Multimedia Appendix 4 [file medinform_v12i1e59273_app4.pdf]

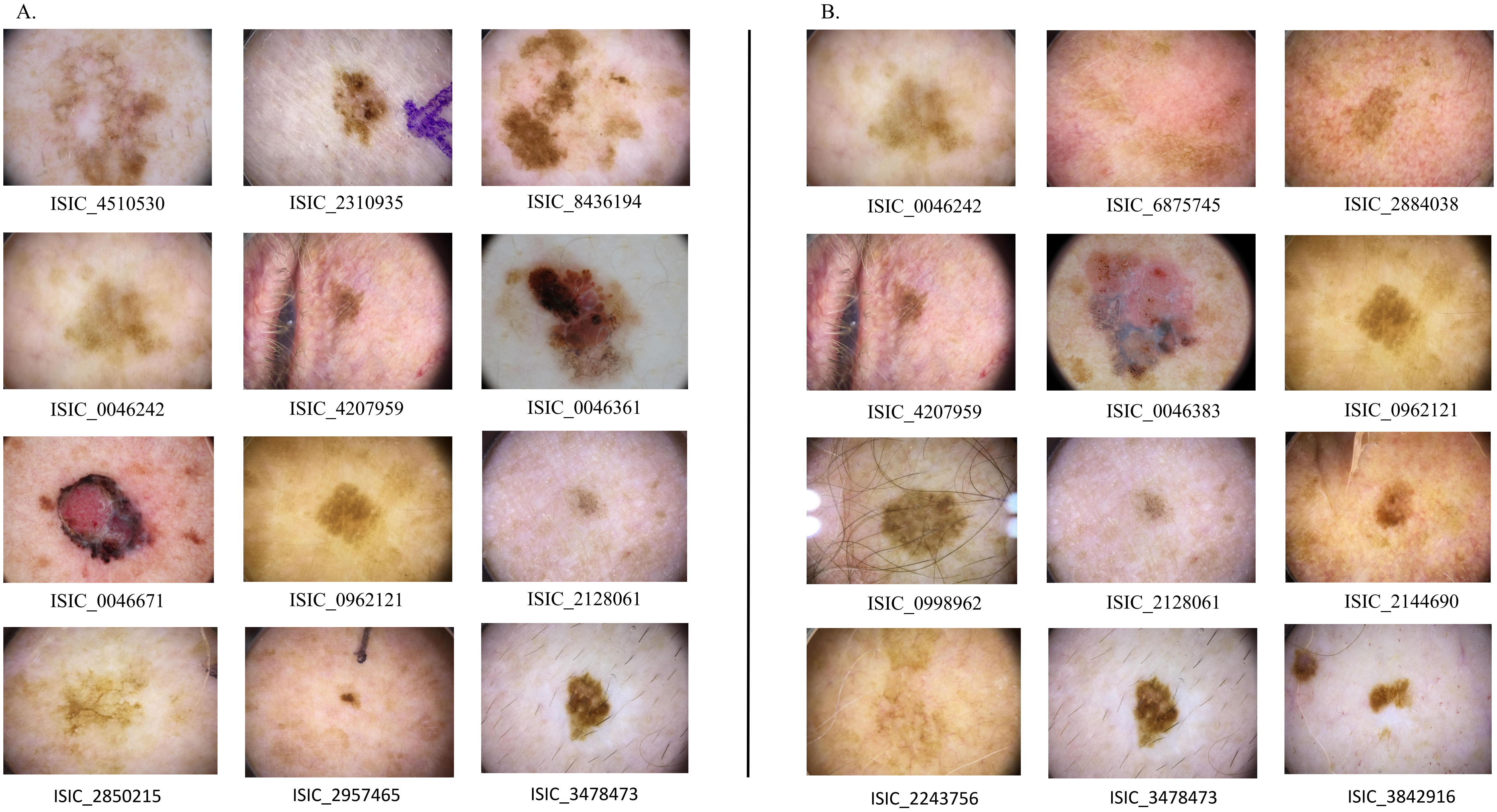
A.
ISIC_4510530
ISIC_2310935
ISIC_8436194
ISIC_0046242
ISIC_4207959
ISIC_0046361
ISIC_0046671
ISIC_0962121
ISIC_2128061
ISIC_2850215
ISIC_2957465
ISIC_3478473
B.
ISIC_0046242
ISIC_6875745
ISIC_2884038
ISIC_4207959
ISIC_0046383
ISIC_0962121
ISIC_0998962
ISIC_2128061
ISIC_2144690
ISIC_2243756
ISIC_3478473
ISIC_3842916

C.

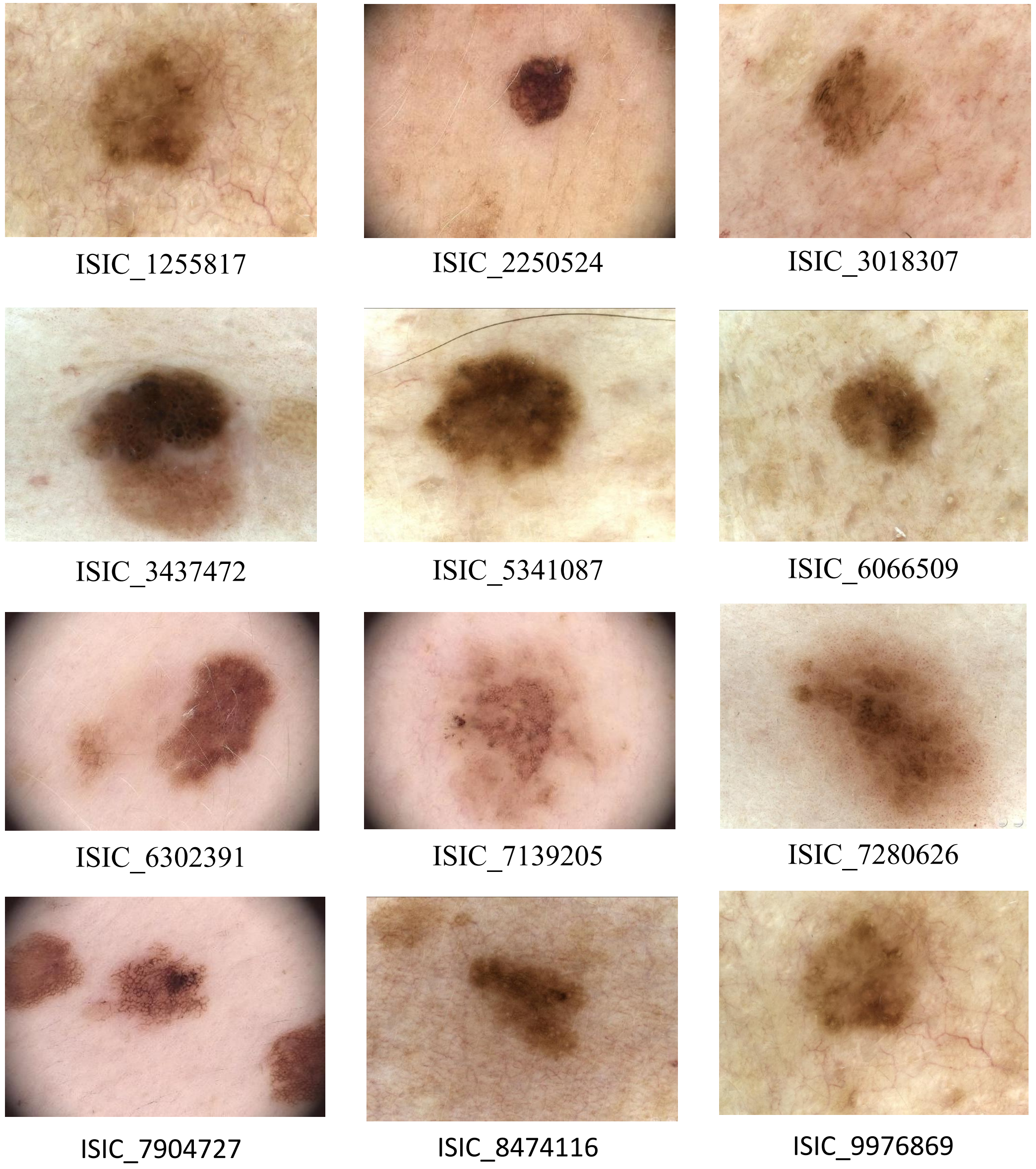
ISIC_1255817
ISIC_2250524
ISIC_3018307
ISIC_3437472
ISIC_5341087
ISIC_6066509
ISIC_6302391
ISIC_7139205
ISIC_7280626
ISIC_7904727
ISIC_8474116
ISIC_9976869

D.

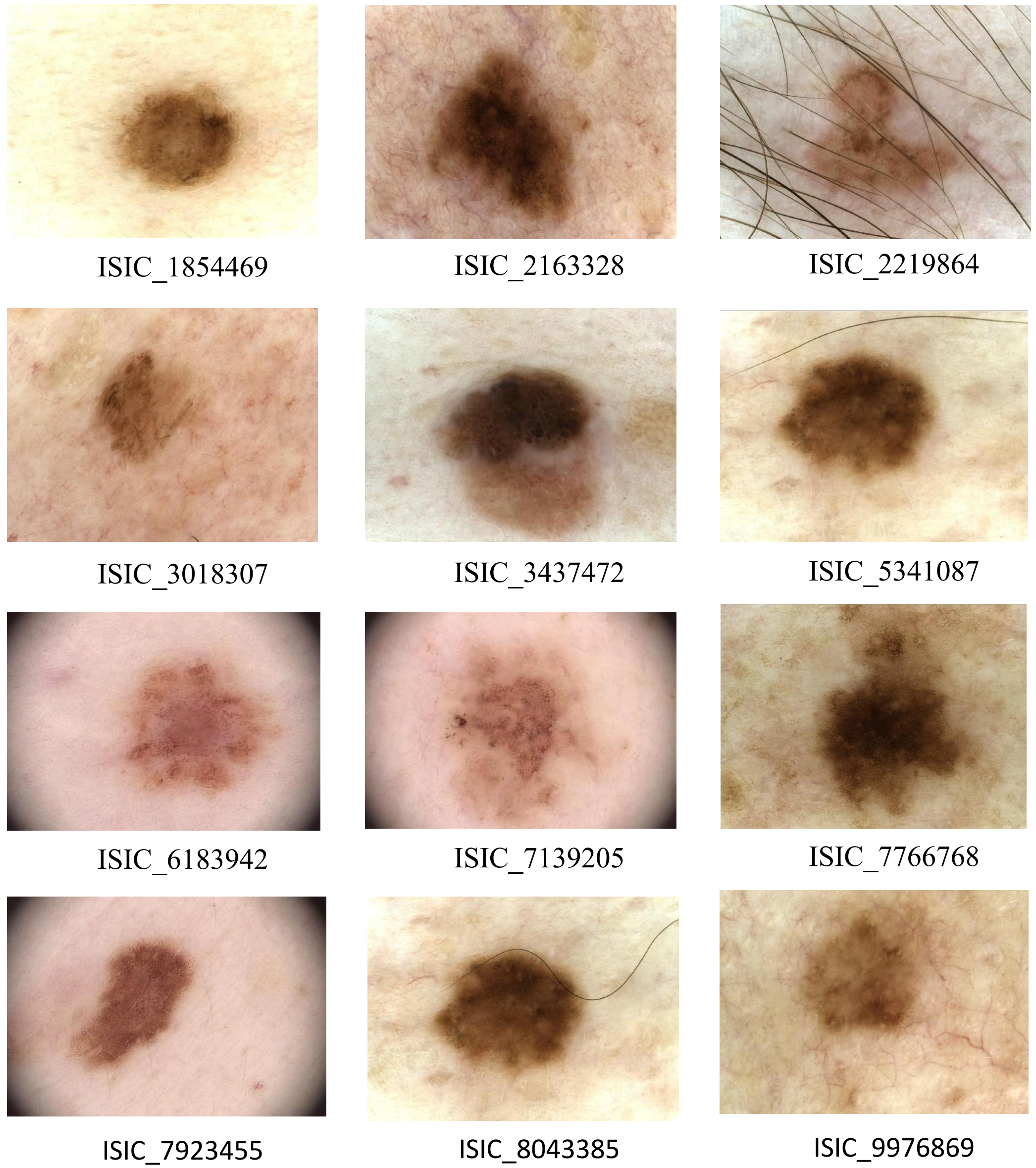
ISIC_1854469
ISIC_2163328
ISIC_2219864
ISIC_3018307
ISIC_3437472
ISIC_5341087
ISIC_6183942
ISIC_7139205
ISIC_7766768
ISIC_7923455
ISIC_8043385
ISIC_9976869
